# Supplementary material for: From the Physicochemical Characteristic of Novel Hesperetin Hydrazone to Its In Vitro Antimicrobial Aspects
Source: Molecules. 2022 Jan 27;27(3):845. doi: 10.3390/molecules27030845 (PMC8839478; doi:10.3390/molecules27030845)
Supplement: Supplementary file 1 [file molecules-27-00845-s001.zip › molecules-1551714-supplementary.pdf]

*Supplementary Materials*

# From the Physicochemical Characteristic of Novel Hesperetin Hydrazone to its In Vitro Antimicrobial Aspects

**Anna Sykuła [1,*], Elżbieta Łodyga-Chruścińska [1], Eugenio Garribba [2], Dorota Kręgiel [3], Aliaksandr Dzeikala [1], Elżbieta Klewicka [4] and Lidia Piekarska-Radzik [4]**

[1] Institute of Natural Products and Cosmetics, Faculty of Biotechnology and Food Sciences, Lodz University of Technology, 90-537 Łódź, Poland; anna.sykula@p.lodz.pl (A.S.), elzbieta.lodyga-chruscinska@p.lodz.pl (E.Ł.-C.); aliaksandr.dzeikala@gmail.com (A.D.)

[2] Department of Medical, Surgical and Experimental Sciences, University of Sassari, Viale San Pietro, I-07100 Sassari, Italy; garribba@uniss.it (E.G.)

[3] Department of Environmental Biotechnology, Faculty of Biotechnology and Food Sciences, Lodz University of Technology, 90-530 Łódź, Poland; dorota.kregiel@p.lodz.pl (D.K.)

[4] Institute of Fermentation Technology and Microbiology, Faculty of Biotechnology and Food Sciences, Lodz University of Technology, 90-530 Łódź, Poland; elzbieta.klewicka@p.lodz.pl (E.K.); lidia.piekarska-radzik@edu.p.lodz.pl (L.P.-R.)

* Correspondence: anna.sykula@p.lodz.pl



Corresponding author: e-mail address: anna.sykula@p.lodz.pl

## 2. Materials and methods

### NMR spectra

**Figure S1**. $^{1}$H NMR (400 MHz) spectrum of HABH in DMSO-d$_6$ at 20 ºC.

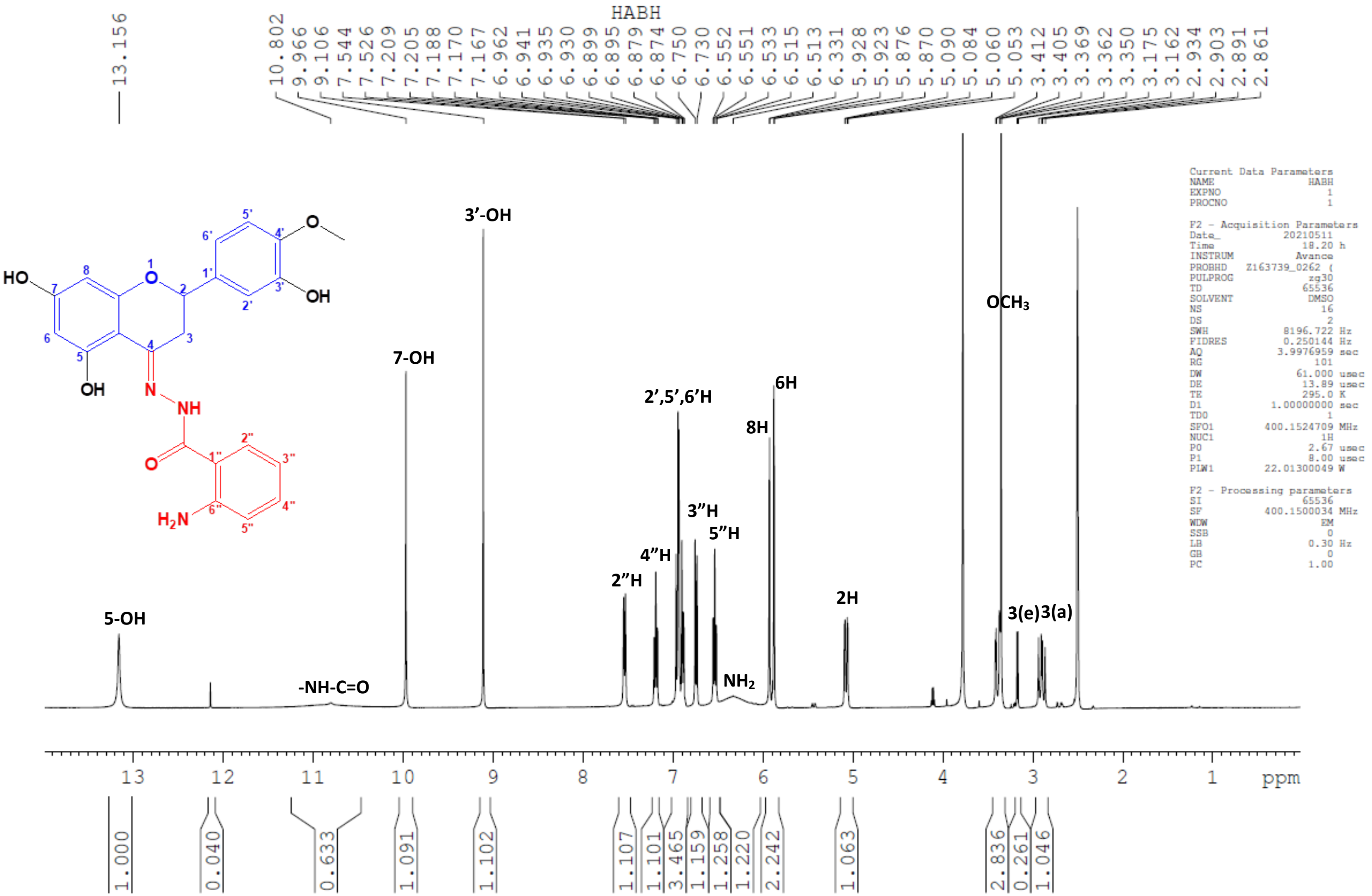

**Figure S2.** $^{13}C$ NMR (400 MHz) spectrum of HABH in DMSO-$d_6$ at 20 ºC.

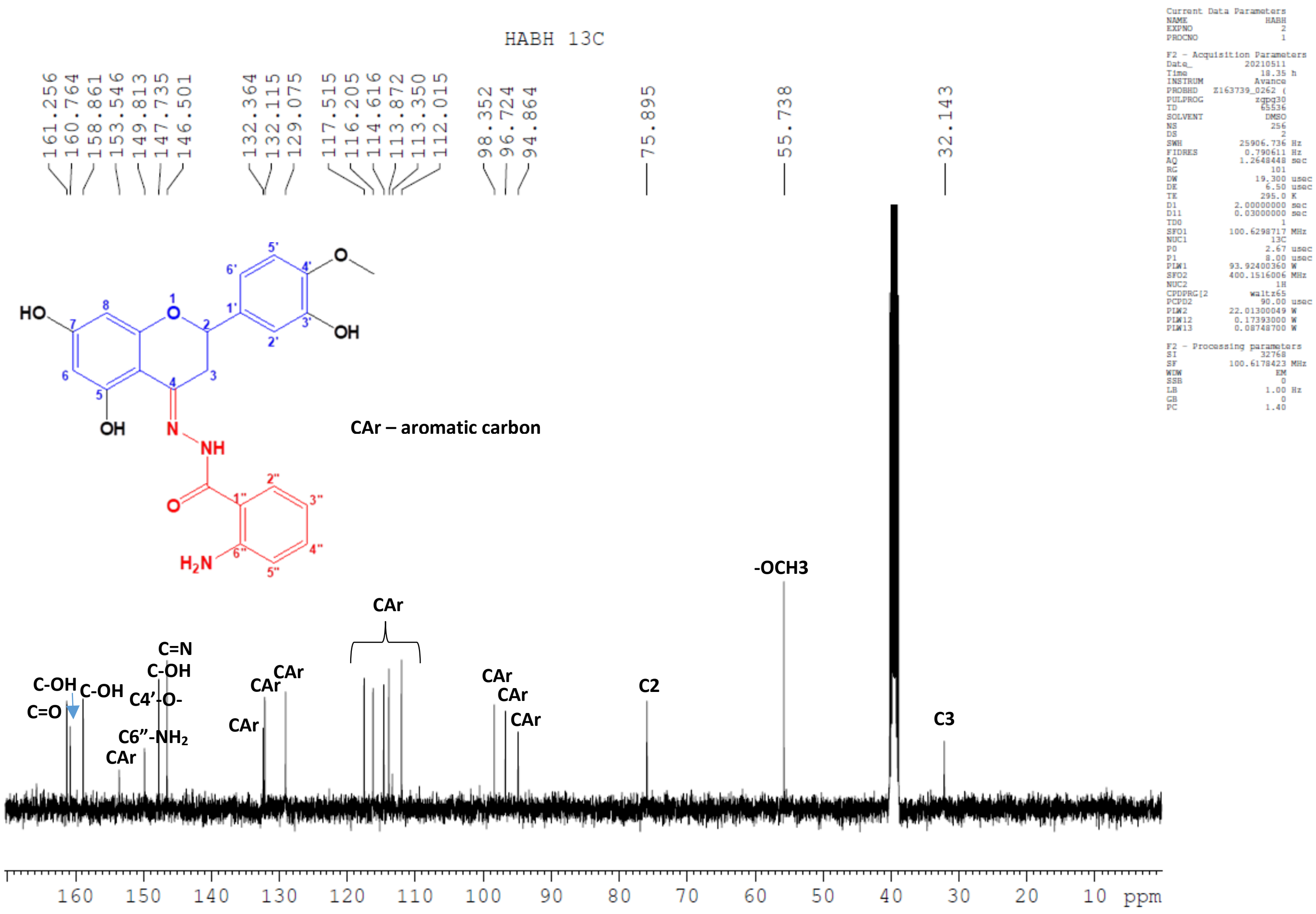




Corresponding author: e-mail address: anna.sykula@p.lodz.pl

**ESI MS spectra**

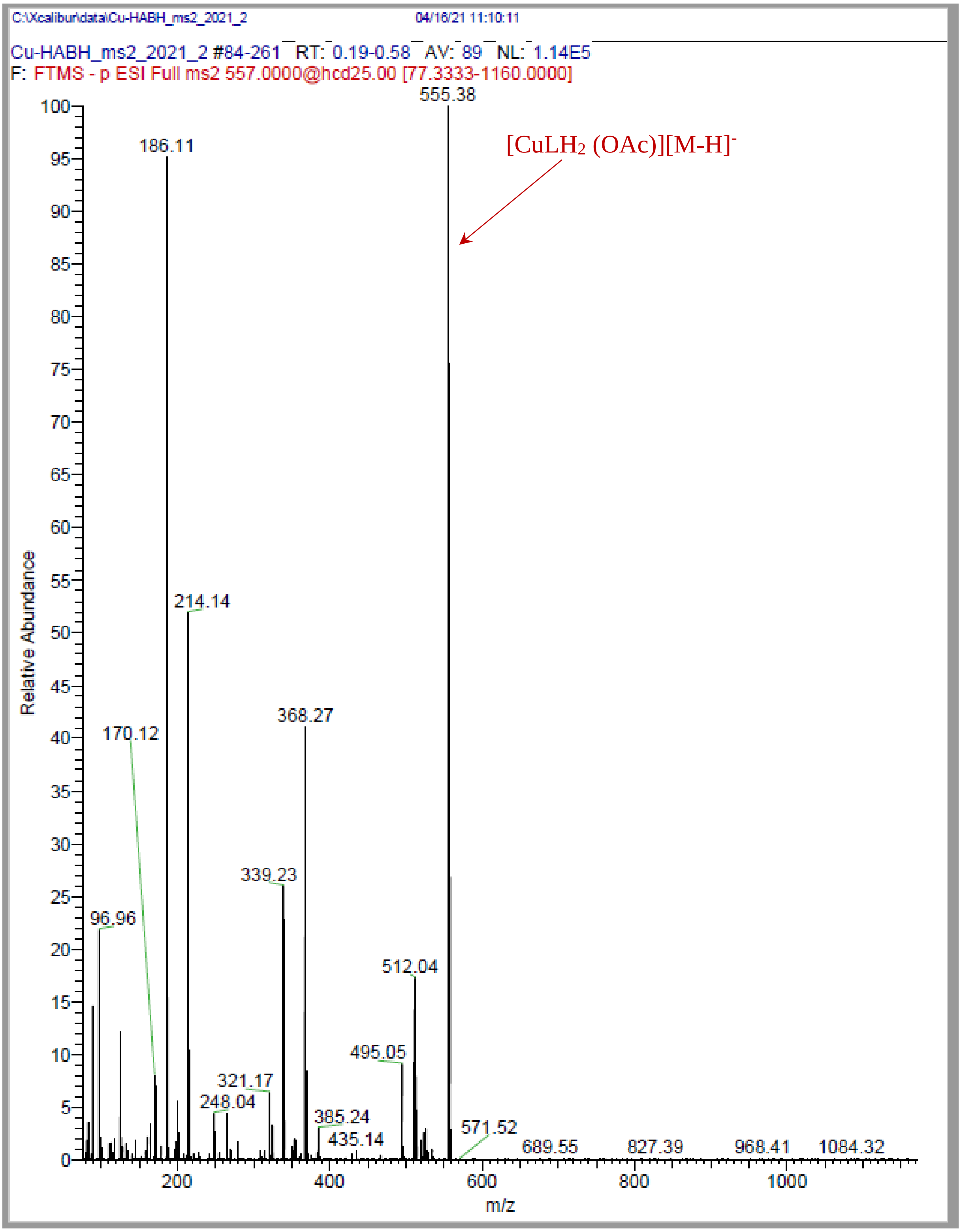


**Figure S3.** Mass spectrum of the CuHABH complex.



Corresponding author: e-mail address: anna.sykula@p.lodz.pl

Potentiometry

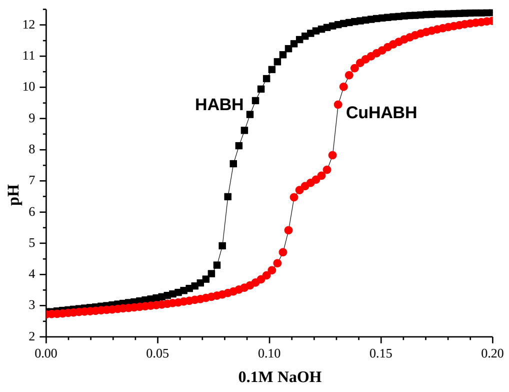


**A**

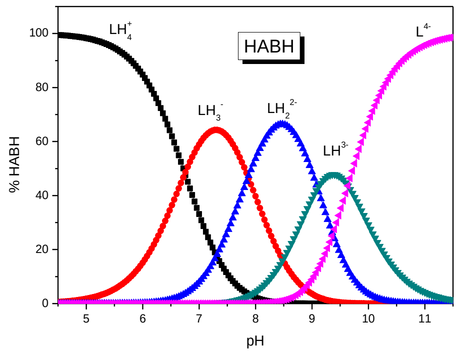


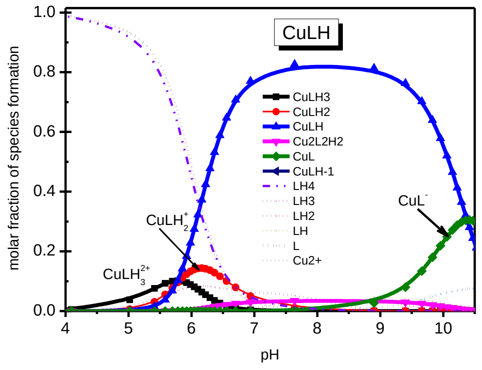


**B** **C**

**Figure S4** (A) Titration curves of HABH and CuHABH systems; (B) Species distribution curves for HABH and (C) Cu(II)–HABH systems. Ligand/metal = 1/1, $C_L = 5 \times 10^{-4}$ M; $C_{Cu(II)} = 5 \times 10^{-4}$ M.



Corresponding author: e-mail address: anna.sykula@p.lodz.pl

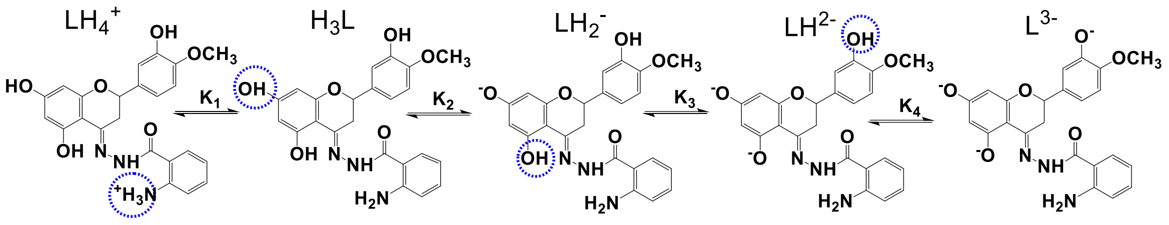


**A**

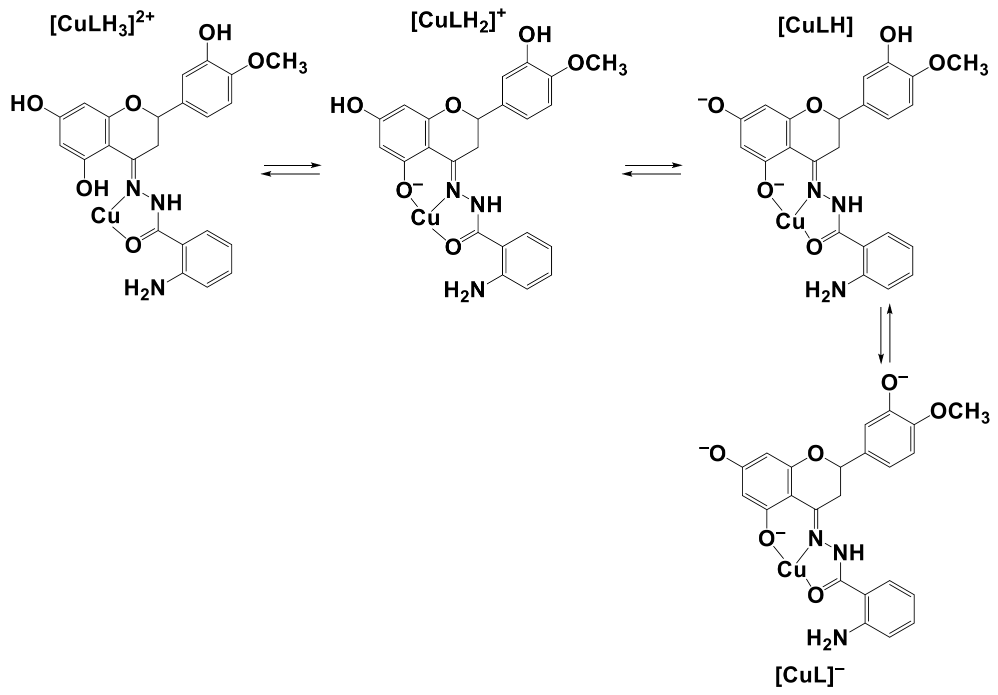


**B**

**Figure S5.** Proposed scheme of protonation/deprotonation equilibria during pH-metric titration of HABH (A) and CuHABH (B). The charge of Cu ions is +2 in the proposed structure of complexes for simplicity it is omitted.



Corresponding author: e-mail address: anna.sykula@p.lodz.pl

## UV-vis spectra

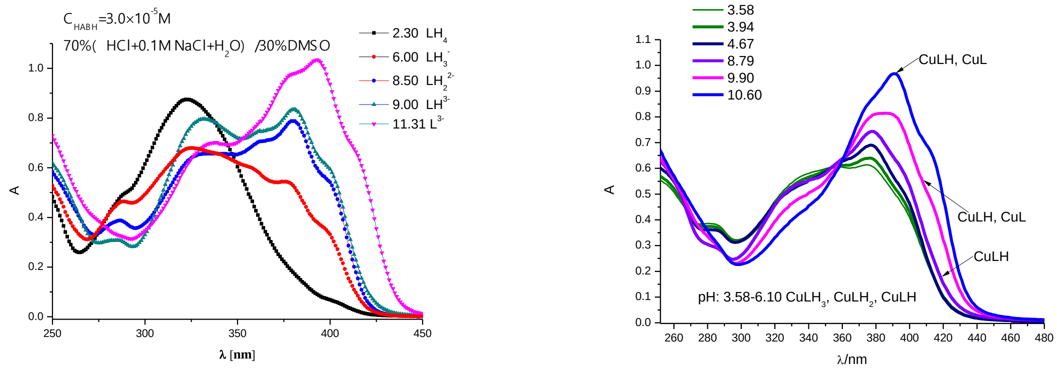


**HABH** **CuHABH**

**Figure S6**. Electronic absorption (UV–vis) spectra recorded in DMSO/water mixture (30%/70% v/v fraction) at different pH corresponding to the dominant species in HABH or CuHABH systems. $C_{HABH}$= $C_{CuHABH}$ = 1 × $10^{-5}$ M. The values of pH are indicated next to the spectra.

## Results and Discussion

IR spectra

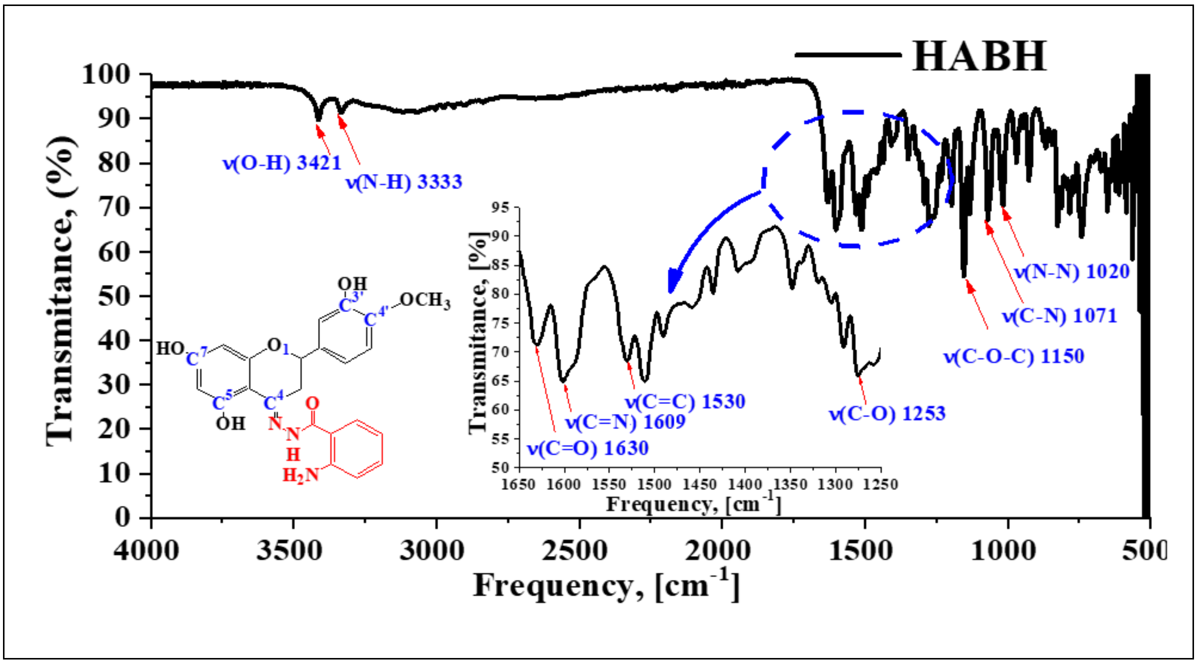




Corresponding author: e-mail address: anna.sykula@p.lodz.pl

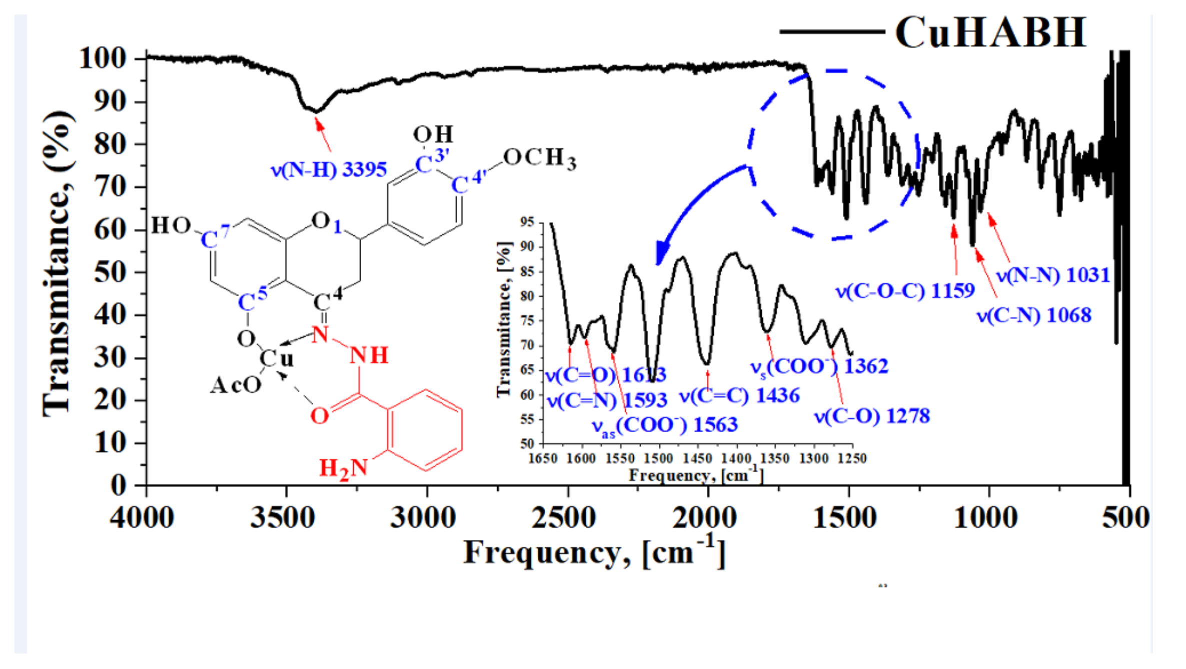


**Figure S7.** FT-IR spectra of ligand HABH and complex CuHABH ([CuLH$_2$(OAc)]).



Corresponding author: e-mail address: anna.sykula@p.lodz.pl

EPR

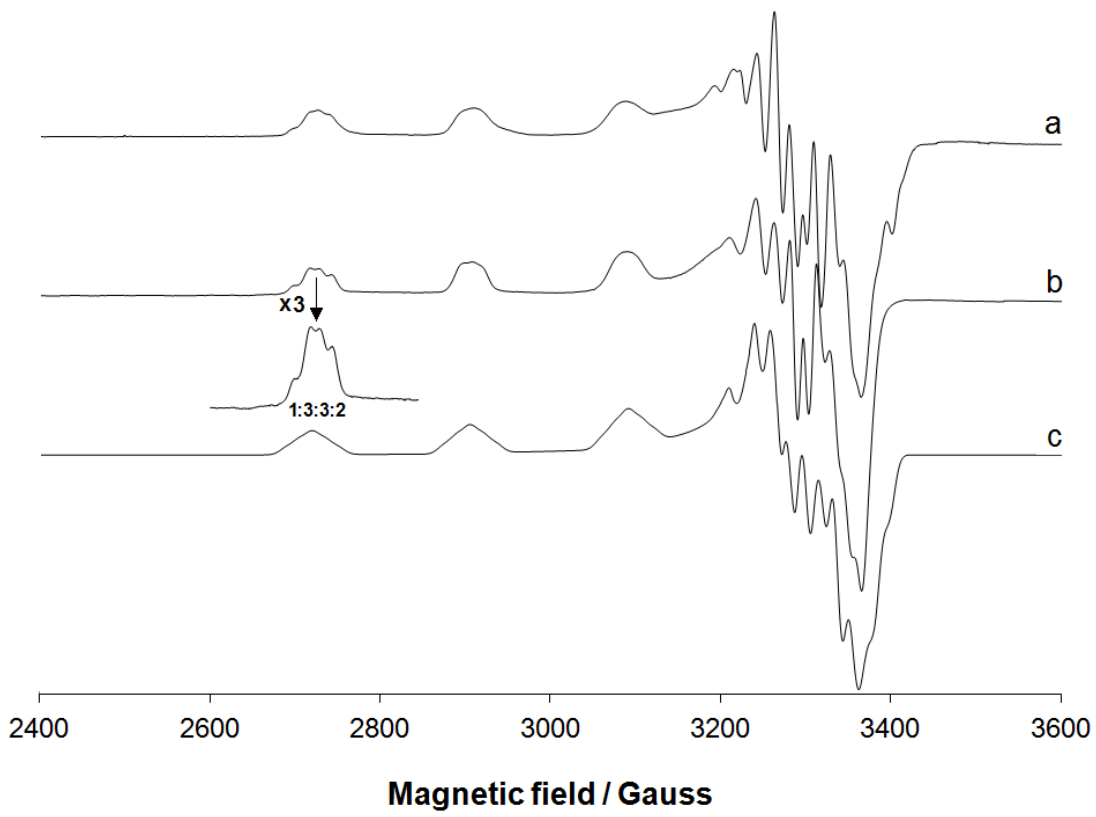


**Figure S8**. EPR spectra recorded at 77 K on the polycrystalline powder of [$CuLH_2(OAc)$] dissolved in: (a) DMF and (b) DMSO. In the trace c the spectrum in DMSO, obtained with WinEPR SimFonia software, is reported.; it was generated using $g_x$ = 2.038, $g_y$ = 2.061, $g_z$ = 2.241, $A_x$(Cu) = -12.0 × $10^{-4}$ $cm^{-1}$, $A_y$(Cu) = -13.0 × $10^{-4}$ $cm^{-1}$, $A_z$(Cu) = -194.0 × $10^{-4}$ $cm^{-1}$, $A_x(^{14}N)$ = $A_y(^{14}N)$ = $A_z(^{14}N)$ = 16.0 × $10^{-4}$ $cm^{-1}$. The first parallel resonance of the spectrum in DMSO is also shown amplifying the experimental signal three times.



Corresponding author: e-mail address: anna.sykula@p.lodz.pl

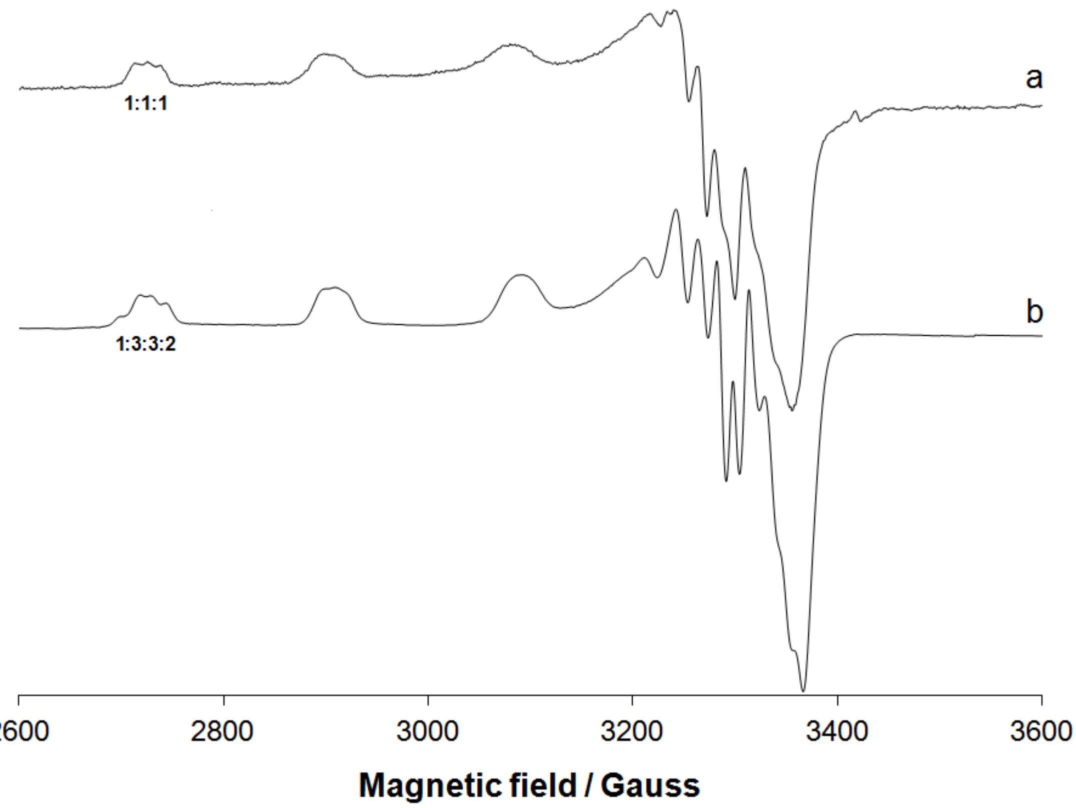


**Figure S9.** Anisotropic EPR spectra recorded at 77 K on: (a) system $^{63}CuSO_4{\cdot}5H_2O$ and HABH as $LH_3$ protonated form in solution (molar ratio 1:1 and $Cu^{II}$ concentration of $1.0 \times 10^{-3}$ M) and (b) solid compound $[CuLH_2(OAc)]$ dissolved in DMSO.



Corresponding author: e-mail address: anna.sykula@p.lodz.pl

## Interaction of the compounds with CT DNA

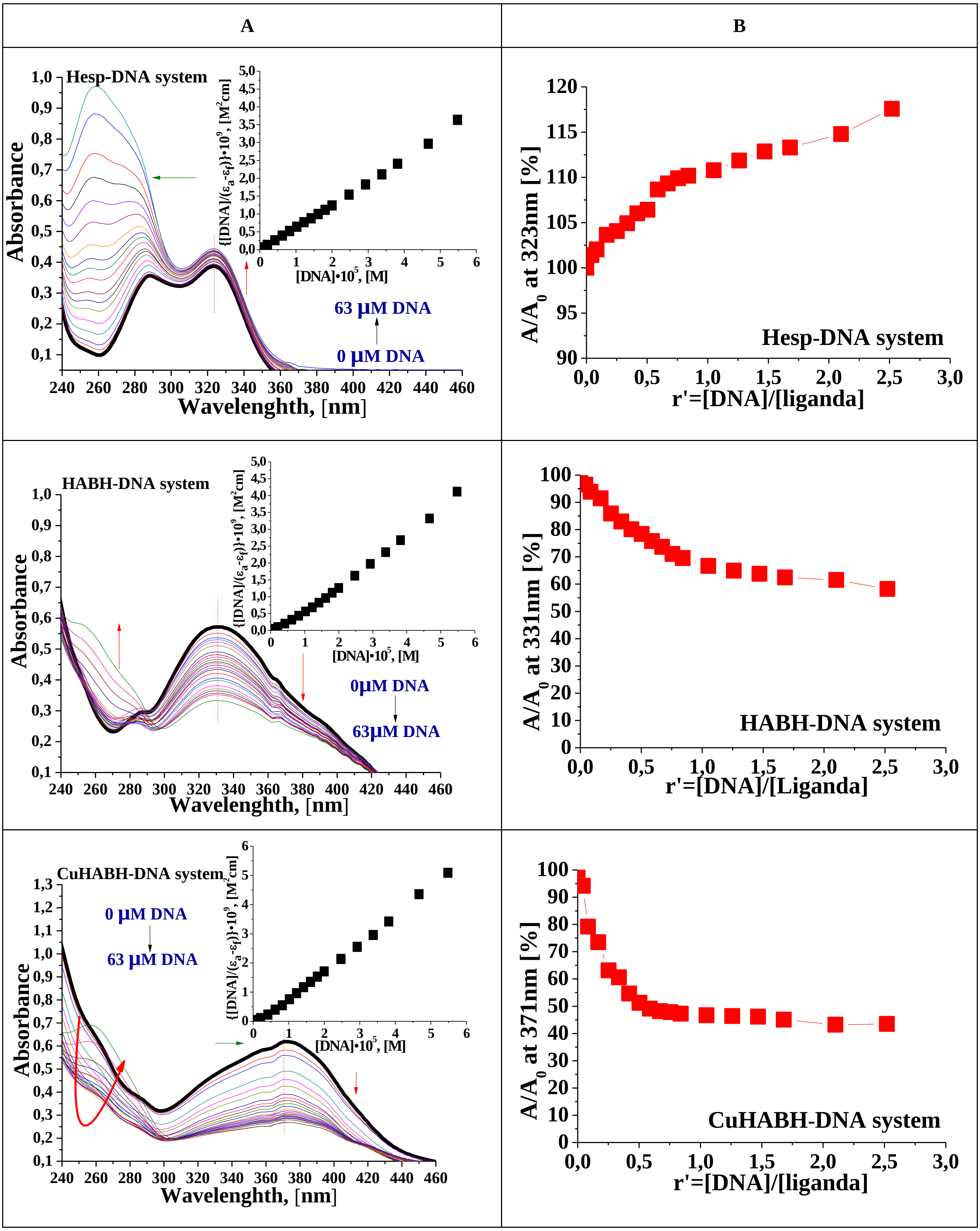


**Figure S10.** (A) Spectrophotometric titration of HESP, HABH, CuHABH with CT DNA solution ($C_{CTDNA}$ = 0–63µM; $C_{compound}$ = 25µM); the arrows show the direction of changes in the spectra. The inset represents the plot of



Corresponding author: e-mail address: anna.sykula@p.lodz.pl

[DNA]/($\varepsilon_a$ - $\varepsilon_f$) vs [DNA]. (B) Effect of test compound concentration on the absorption intensity of compound-CT DNA system.



Corresponding author: e-mail address: anna.sykula@p.lodz.pl

## Competitive study with TO

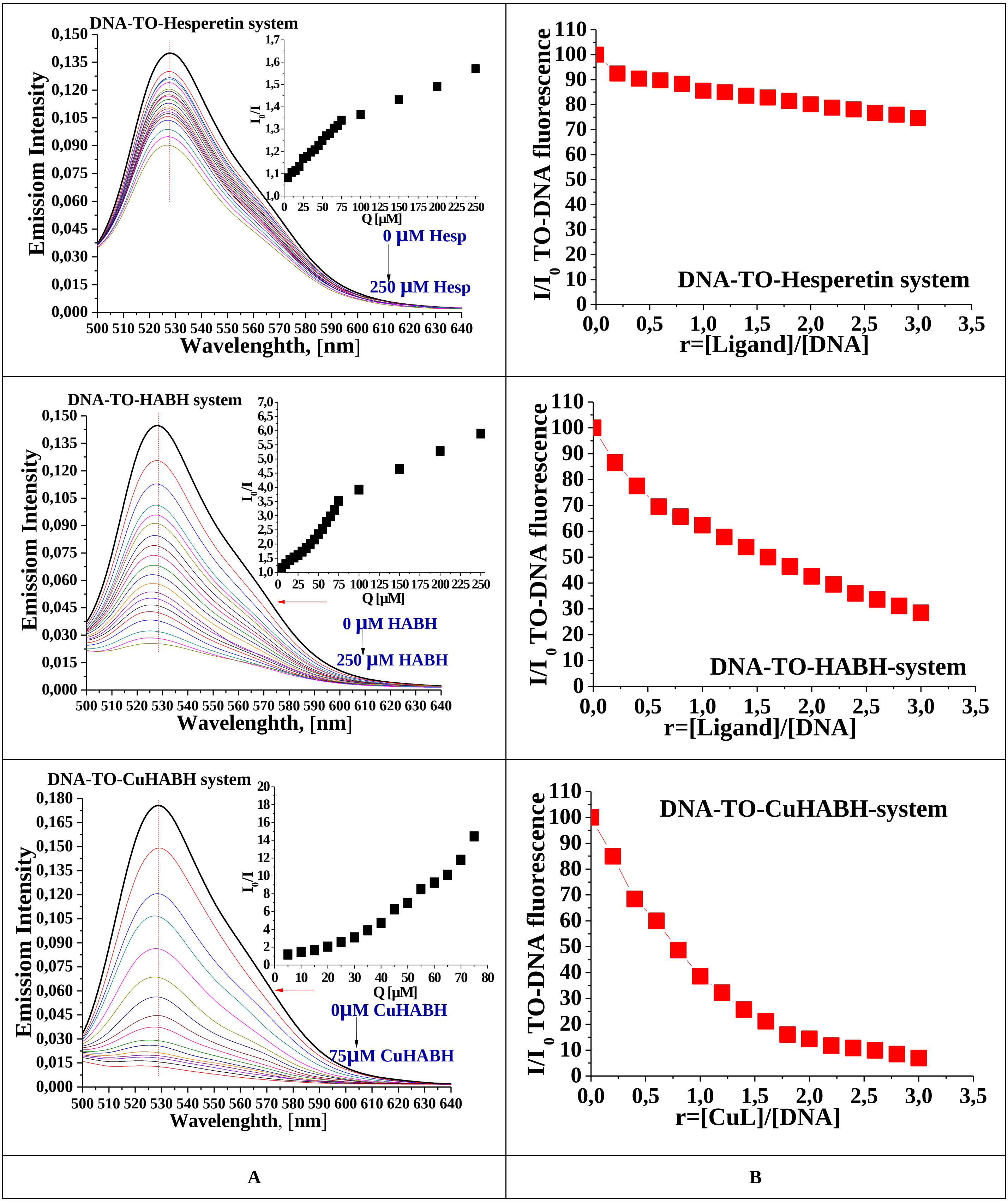


**Figure S11** (**A**) Emission spectra of HESP, HABH and CuHABH with increasing concentration of CT DNA (C = 0–75 μM for CuHABH or up to 250 for HESP, HABH; $C_{CT\ DNA}$ = 25μM); arrows show the direction of changes. Inset:



Corresponding author: e-mail address: anna.sykula@p.lodz.pl

Stern-Volmer relationship $I_0/I$ versus [DNA]. (**B**) Relative fluorescence intensity (I/Io, %) at $\lambda_{em}$ = 527 nm versus *r* (=[compound]/[DNA]) in solution of CT DNA-TO adduct.



Corresponding author: e-mail address: anna.sykula@p.lodz.pl
